# Two Opposing Faces of Retinoic Acid: Induction of Stemness or Induction of Differentiation Depending on Cell-Type

**DOI:** 10.3390/biom9100567

**Published:** 2019-10-04

**Authors:** Belén Mezquita, Cristóbal Mezquita

**Affiliations:** 1 Departament de Biomedicina, Laboratori de Genètica Molecular, Universitat de Barcelona, 08036 Barcelona, Spain; belenmezquita@ub.edu; 2 Institut d’Investigacions Biomèdiques August Pi i Sunyer (IDIBAPS), 08036 Barcelona, Spain; 3 Departament de Ciències Bàsiques, Universitat Internacional de Catalunya, 08195 Sant Cugat del Vallès, Spain

**Keywords:** All-trans retinoic acid (ATRA), stemness, differentiation, regenerative medicine, cancer

## Abstract

Stem cells have the capacity of self-renewal and, through proliferation and differentiation, are responsible for the embryonic development, postnatal development, and the regeneration of tissues in the adult organism. Cancer stem cells, analogous to the physiological stem cells, have the capacity of self-renewal and may account for growth and recurrence of tumors. Development and regeneration of healthy tissues and tumors depend on the balance of different genomic and nongenomic signaling pathways that regulate stem cell quiescence, proliferation, and differentiation. During evolution, this balance became dependent on all-trans retinoic acid (RA), a molecule derived from the environmental factor vitamin A. Here we summarize some recent findings on the prominent role of RA on the proliferation of stem and progenitor cells, in addition to its well-known function as an inductor of cell differentiation. A better understanding of the regulatory mechanisms of stemness and cell differentiation by RA may improve the therapeutic options of this molecule in regenerative medicine and cancer.

## 1. Introduction

Retinoic acid (RA) regulates a wide range of biological processes during development and in adult organisms [1,2,3,4,5,6,7,8,9]. Retinoic acid signaling is dependent on cells that can metabolize vitamin A (retinol) to RA. Retinol dehydrogenases oxidize retinol to retinal, and aldehyde dehydrogenases (ALDH1A1, ALDH1A2, and ALDH1A3) oxidize retinal to RA [2,10]. Retinoic acid released from these cells generates gradients that regulate neighboring cells. The precise RA level depends on the availability of vitamin A (retinol), the activity of enzymes involved in RA biosynthesis (retinol dehydrogenases and aldehyde dehydrogenases), and the RA catabolism by CYP26 enzymes [11,12,13]. 

Retinoic acid regulates transcription by interacting with heterodimers of nuclear RA receptors (RARα, RARβ, and RARγ) and retinoid X receptors (RXRα, RXRβ, and RXRγ) bound to RA response elements (RAREs) in the promoters of target genes [4,14,15]. The expression of over 500 genes is upregulated or downregulated by RA [16]. Moreover, RA controls other transcriptional signaling pathways via different nuclear receptors, such as the peroxisome proliferator-activated receptor β/δ [17,18], and can also regulate different protein kinases in a nontranscriptional fashion [19,20,21]. 

Although RA has been widely described as an inductor of cell differentiation, depending on cell-type, RA can antagonize cell differentiation and promote stemness (Table 1).

## 2. Retinoic Acid Induces Stemness or Differentiation in the Mammary Gland and Breast Cancer Cells

Unlike other organs, the mammary gland tissue undergoes development postnatally. An adequate balance between stem self-renewal and stem cell differentiation is essential for this process. Prodifferentiation and antidifferentiation effects of RA have been reported during mammary gland development and breast cancer [23,24,37].

### 2.1. Growth-Promoting and Growth-Inhibiting Actions of RA in Breast Cancer Depend on the Cell Context-Specific Balance of Activation of Transcriptional and Nontranscriptional Pathways

**Figure 1:**
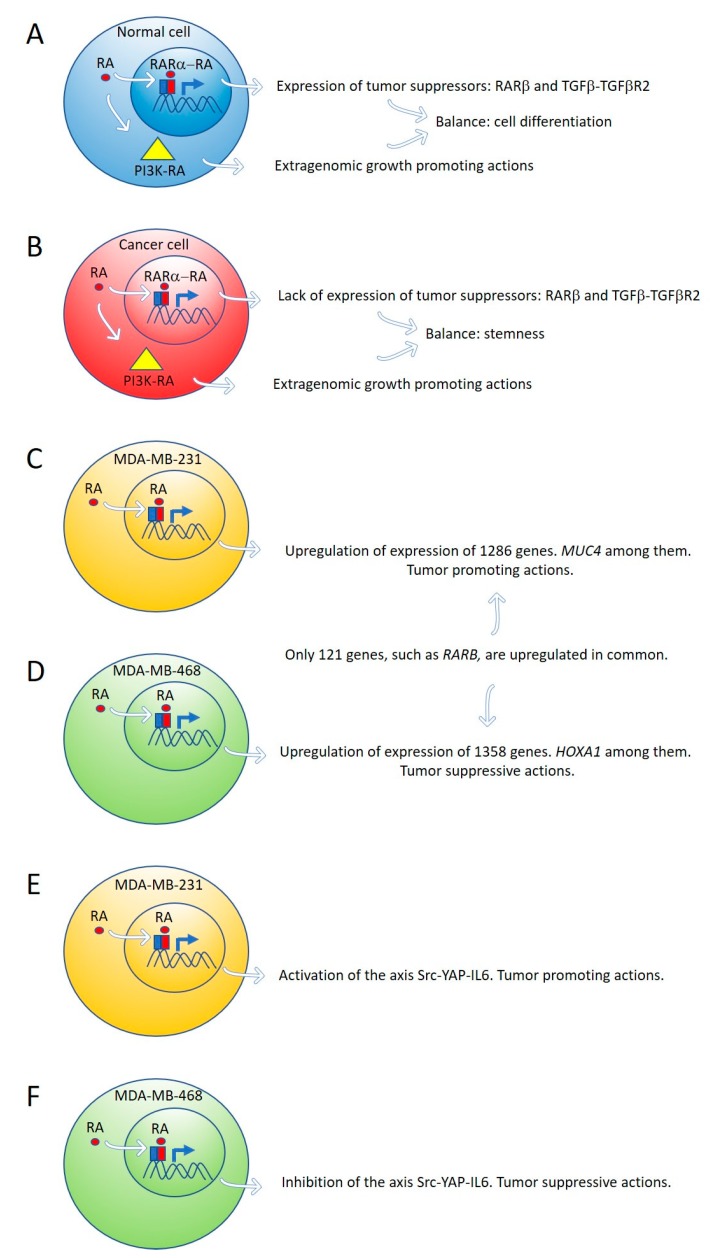
Expression of tumor suppressor genes by retinoic acid (RA) in healthy cells counteracts the growth-promoting activity of nontranscriptional RA signaling pathways, such as PI3K-AKT (**A**). In cancer cells, lack of expression of tumor suppressor genes by RA cannot counteract the extragenomic tumor-promoting actions of RA (**B**). Differential gene expression induced by ALDH1A3 or RA in MDA-MB-231 and MDA-MB-468 cells (**C**,**D**). Retinoic acid upregulates the signaling pathway Src-YAP-IL6 involved in stemness in triple-negative MDA-MB-231 breast cancer cells (**E**) and downregulates the same pathway in triple-negative MDA-MB-468 breast cancer cells (**F**).

By global gene expression microarray analysis, Rossetti et al. [23] determined that in breast cancer cells (T47D^Ctrl^) grown under “physiological” RA culture conditions, many RARα-target genes, coding for tumor suppressor signaling pathways, as RARβ and the TGFβ-TGFβR2, are in a repressed transcriptional state marked by epigenetic histone modifications. In this situation, lack of expression of tumor suppressor genes cannot counteract the growth-promoting activity of nontranscriptional signaling pathways such as PI3K-AKT, triggered by direct interaction of RARα and the catalytic subunit of PI3K [23]. The degree of inhibition of RARα transcriptional function is variable in different breast cancer cell lines: mild in T47D^Ctrl^, severe in T47D^G303E^, and extremely severe in T47D^403^. Both in vitro and in vivo treatment with supraphysiological doses of exogenous RA significantly promoted T47D^403^ breast cancer cell invasion [23] (Figure 1A,B). 

### 2.2. Retinoic Acid Induces Tumor-Promoting or Tumor-Suppressive Actions in Triple-Negative Breast Cancer Cells Due to Variable Gene Expression in Cell Lines with Differences in DNA Methylation

Marcato et al. [24] reported that the effects of RA and ALDH1A3 activity were tumor-promoting in MDA-MB-231 and MDA-MB-435 triple-negative breast cancer cells, but tumor-suppressive in triple-negative MDA-MB-468 breast cancer cells. The opposing tumor growth effects of ALDH1A3/RA in breast cancer cells depend upon differential gene expression induced by ALDH1A3 or RA in MDA-MB-231 and MDA-MB-468 cells. Increased ALDH1A3 expression upregulated 1286 and 1358 genes in MDA-MB-231 and MDA-MB-468 cells, respectively. A large divergence in gene expression changes induced by ALDH1A3 in the two cell lines was observed because only 121 genes were upregulated in common in both cell lines. *RARβ* is one of these genes (Figure 1C,D). 

One of the ALDH1A3-induced genes in MDA-MB-468 cells is the homeobox transcription factor A1 (HOXA1). The promotor of *HOXA1* possesses a RARE sequence that was previously shown to be inducible by RA [38]. HOXA1 expression is significantly reduced by ALDH1A3 knockdown and induced by RA in MDA-MB-468 cells but is undetectable in MDA-MB-231 cells [24]. *HOXA1* is hypermethylated in MDA-MB-231 cells and hypomethylated in MDA-MB-468 cells [24]. *HOXA1* is often hypermethylated in cancer, suggesting a tumor-suppressive function [39,40].

Mucin 4 (*MUC4*), a potential oncogene with a RARE, inducible by RA, and associated with triple-negative breast cancer [41,42], is significantly induced by ALDH1A3 and RA in MDA-MB-231 cells, but not in MDA-MB-468 cells. *MUC4* is hypermethylated in MDA-MB-468 and hypomethylated in MDA-MB-231 [24]. MUC4 is typically hypomethylated in cancers, and its expression is associated with more aggressive cancer [41,42,43,44,45]. *MUC4* knockdown in MDA-MB-231 cells reduced their tumorigenic and metastatic properties [42], suggesting *MUC4* may represent a gene that contributes to ALDH1A3/RA-mediated tumor growth and metastasis of MDA-MB-231 cells [24]. 

### 2.3. Retinoic Acid Upregulates the Signaling Pathway Src-YAP-IL6 Involved in Stemness in Triple-Negative MDA-MB-231 Breast Cancer Cells and Downregulates the Same Pathway in Triple-Negative MDA-MB-468 Breast Cancer Cell Line

Retinoic acid induces tumor suppression in tumor xenografts of MDA-MB-468 breast cancer cells while increasing tumor growth and metastasis in xenografts of MDA-MB-231 [24]. We have used these triple-negative breast cancer cell lines as a research model to investigate the role of RA on the regulation of the signaling pathway Src-YAP-Interleukin 6 involved in stemness [25]. We found that RA activates this pro-invasive axis in triple-negative MDA-MB-231 breast cancer cells, yielding to an increased invasion of these cells. On the contrary, RA inhibits the Src-YAP-IL6 axis of triple-negative MDA-MB-468 cells, which results in decreased invasion phenotype (Figure 1E,F). In both types of cells, inhibition of the Src-YAP-IL6 axis by the Src inhibitor PP2 drastically reduces migration and invasion. The Src-YAP-IL6 axis controls invasion, metastasis, resistance to therapy, and stemness of MDA-MB-231 breast cancer cells [46,47]. IL-6 is the first universal transcriptional target of YAP involved in promoting stemness conserved from flies to humans [46,48]. 

Overexpression of IL-6 induces cancer cell proliferation, angiogenesis, and metastasis through stimulating STAT3, MAPK, and Akt signaling pathways [49]. IL-6 regulates cancer stem cell, mesenchymal stem cell formation, and epithelial to mesenchymal transition in cancer, and is a contributing factor for chemoresistance [49]. Sansone et al. [50] found that IL-6 mRNA was robustly elevated in mammospheres compared with breast epithelium and was required for their self-renewal and aggressive potential. Autocrine IL6-STAT3 signaling increases stem cell properties with efficient tumor colonization and outgrowth in vivo. Conversely, blockage of IL-6 reduces tumor burden and metastasis [51,52,53,54]. 

Nuclear YAP phosphorylation in MDA-MB-231 breast cancer cells depends on Src activity. Until recently, activation of YAP was believed to solely depend on the inhibition of the Hippo signaling pathway that retains YAP in the cytoplasm [55]. To assess if YAP activation in MDA-MB-231 breast cancer cells depends on Src activity, as observed in other cancer cells [56,57,58], we used Src inhibition by PP2, Src interference by siRNA and transfection of Src into MDA-MB-231 breast cancer cells. Src inhibition by PP2 and Src interference decreased YAP activity and downregulated IL-6 expression, while Src transfection activated YAP and upregulated IL-6 [25]. 

The mechanism of Src activation induced by RA is not known at present. Mechanisms independent of transcription have been reported in breast cancer cells [23]. However, the activation of the Src-YAP-IL6 axis we have observed should be the consequence of a genomic action of RA, given the 48 h delay following incubation with supraphysiological concentrations of RA (5 μM). Extragenomic effects of RA in breast cancer cells are produced faster and with lower levels of RA [23].

Overexpression of MUC4 in triple-negative breast cancer cells induced by RA [24] is an attractive candidate for Src activation because cell knockdown of MUC4 in pancreatic carcinoma decreased Src tyrosine phosphorylation significantly [59]. IL-6 induces MUC4 expression through the gp130-STAT3 pathway in gastric cancer cell lines [60].

An association of YAP activity and RA signaling with an increase in migration also has been observed in human neural crest cells [61]. YAP, as well as its paralog TAZ, is known to act as a stemness-promoting factor in several tissue types, including hepatic, intestinal, and skin stem cell niches [62,63,64,65]. 

It has been reported that MDA-MB-231 and MDA-MB-468 are non-sphere-forming cells lines [66]. However, it is not known how the presence of RA could affect mammosphere formation of these cell lines [24] and whether these in vitro assays may reflect the expansion of breast cancer stem and nonstem cells in vivo. Using tumor xenografts, RA increases tumor growth and metastasis of MDA-MB-231 and decreases tumor growth of MDA-MB-468 cells [24].

### 2.4. Retinoic Acid Conferred Stemness Properties to Breast Cancer MCF-7 Cells

**Figure 2:**
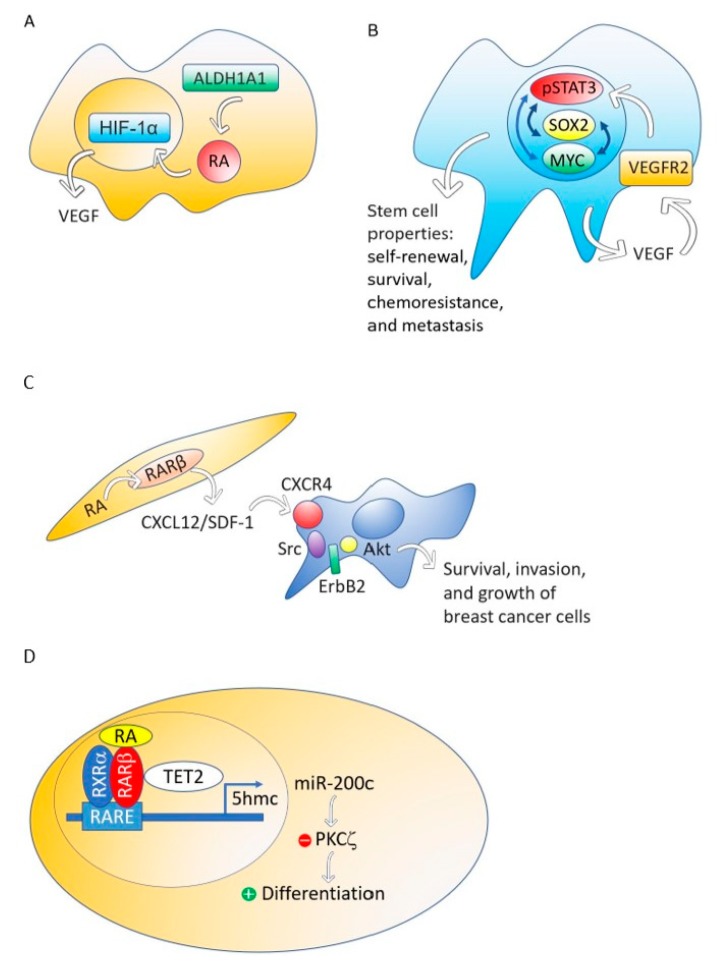
The ALDH1A1-retinoic acid-HIF-1α-VEGF pathway is activated in breast cancer MCF-7 cells (**A**). VEGF drives breast and lung cancer-initiating stem cells through the VEGFR-2-STAT3 signaling pathway that upregulates *MYC* and *SOX2* gene expression (**B**). RA, through RARβ, increases the production of CXCL12/SDF-1 in stroma cells and, consequently, activates the Src-ErbB2-Akt signaling pathway in breast cancer cells, promoting survival, cell growth, and invasion (**C**). When the nontumorigenic, immortalized mammary epithelial cell line, MCF12A and the non-invasive breast cancer cell line T47D were treated with RA, the RA nuclear receptor RARβ associated with a methylcytosine dioxygenase (TET2) that produces DNA demethylation. The consequence is the induction of genes involved in cell differentiation and the activation of miR-200c expression. MiR-200c downregulates stemness targeting the protein kinase PKCζ. RA does not perform these actions in aggressive breast cancers (**D**).

Although different breast cancer cell lines such as 184A1, SUM149, SUM159, and HCC1954 treated with RA presented a decrease in mammosphere formation [67], the breast cancer MCF-7 cell line responds to RA with an increase of stemness through an ALDH1A1-retinoic acid-HIF-1α-VEGF pathway [26] (Figure 2A). It has been reported that VEGF drives breast and lung cancer-initiating stem cells through the VEGFR-2-STATt3 signaling pathway that upregulates MYC and SOX2 [68,69] (Figure 2B). VEGF contributes to the acquisition of stem cell properties, including self-renewal, survival, and chemoresistance through VEGFR2 receptors, VEGF neuropilin receptors [70,71] and intracrine VEGF receptors [72,73]. 

### 2.5. RARβ Expression in the Mammary Gland Stroma Shapes the Tumor Microenvironment Favoring Breast Tumor Growth and Invasion

Although RARβ possesses, in breast cancer cells, many of the functional characteristics of a tumor suppressor, RARβ in the tumor stroma has a dominant role in promoting the growth and progression of mammary epithelial tumors [74]. The mechanism through which stromal RARβ achieves its tumor-promoting effect probably involves the production of CXCL12/SDF-1 in stroma cells and the consequent activation of the Src-ErbB2-Akt signaling pathway in the breast cancer cells (Figure 2C).

### 2.6. Retinoic Acid Induces Cell Differentiation and Downregulates Stemness in a Nontumorigenic Immortalized Mammary Epithelial Cell Line and a Non-Invasive Breast Cancer Cell line but Does Not Perform These Actions in Aggressive Breast Cancers

Using MCF12A, a nontumorigenic immortalized mammary epithelial cell line, or T47D, a non-invasive breast cancer cell line, RA induces genes involved in cell differentiation such as *RUNX1, BMP6, IKZF1* and *CAV1,* and activates the expression of noncoding RNAs that downregulate stemness, such as miR-200c [27]. This miRNA targets and suppresses the protein kinase PKCζ, a protein that has a pivotal role in directing the asymmetric division of mammalian stem cells to sustain the stem cell pool [75,76,77]. PKCζ overexpression promotes breast cancer invasiveness and metastasis [78]. However, the triple-negative breast cancer cell line MDA-MB-231 does not respond with cell differentiation and downregulation of stemness to RA treatment [27]. 

Retinoic acid treatment of the nontumorigenic, immortalized mammary epithelial cell line, MCF12A and the non-invasive breast cancer cell line T47D induces the association of the RA nuclear receptor RARβ with a methylcytosine dioxygenase (TET2) [27]. The TET protein family has a crucial role in DNA demethylation by catalyzing the conversion of the modified genomic base 5-methylcytosine into 5-hydroxymethylcytosine (5hmC), thereby activating the target gene expression [79]. Expression of TET2 occurs in the nontumorigenic mammary epithelial cell line MCF12A and also in the non-invasive breast cancer cell line T47D, but repression occurs in aggressive breast cancers [27] (Figure 2D).

Retinoic acid enhanced the nuclear localization of RARβ and TET2, whereas knockdown of RARβ blocked RA mediated TET2 nuclear localization and substantially increased TET2 in the cytoplasm fraction. In contrast to nontumorigenic MCF12A and non-invasive breast cancer cell line T47D, TET2 was predominantly localized in the cytoplasm in aggressive triple-negative breast cancer cell line MDA-MB-231, which is deficient in endogenous RARβ expression. Re-expression of RARβ in MDA-MB-231 cells relocalized TET2 to the nucleus, and the nuclear TET2 level was further enhanced by RA treatment [27].

### 2.7. Retinoic Acid Blocks the Progesterone Induction of Cytokeratin-5 Expressing Breast Cancer Stem Cells

Half of estrogen receptor-positive breast cancers contain a subpopulation of cytokeratin-5 expressing cells that are therapy-resistant and exhibit increased cancer stem cell properties induced by progesterone. Retinoic acid, through RARα or RARγ, blocks progesterone induction of cytokeratin-5 expression and stemness [80]. 

## 3. Janus Faces of RA in Other Tissues

Promotion of either stemness or cell differentiation by the RA signaling pathway also has been observed in pluripotent stem cells, the neural system, the hematopoietic system, colorectal cancer, hepatocellular carcinoma, pancreatic cancer, ovarian cancer, spermatogenesis, and regeneration of tissues, among others. 

### 3.1. Retinoic Acid Sustains Pluripotency and Suppresses Differentiation of Human Induced Pluripotent Stem Cells

Short-time treatment (24 h) with 0.5 μM RA antagonizes cell differentiation sustaining and improving pluripotency. In these conditions, RA inhibits the Wnt canonical pathway and positively modulates the Akt-mTOR signaling pathway. 

### 3.2. Retinoic Acid Induces Stemness or Differentiation in the Neural System

Although RA facilitates differentiation of neurons at the expense of proliferation during neurogenesis [81,82], recent studies have revealed that RA induces proliferation in early neurogenesis in the developing mouse cerebral cortex [83], in the adult hippocampus [28], and also in stem-like glioma cells [29].

#### 3.2.1. Retinoic Acid Induces Proliferation in Cerebral Cortex Early Neurogenesis

Cerebral cortex early neurogenesis is achieved by a proper balance between proliferation and differentiation of progenitor cells. The first progenitors formed in the cortical plate are radial glial cells which generate neurons either directly, or through an indirect mechanism involving the production of intermediate neuronal progenitors, which then give rise to neurons. In the absence of RA, the radial glial cells proliferate less and prematurely produce neurons, leading to their depletion. Furthermore, the lack of RA impairs the generation of intermediate neuronal progenitors, producing a deficit in projection neurons and microcephaly [83]. 

#### 3.2.2. Retinoic Acid Induces Proliferation in Adult Neurogenesis in the Hippocampus

Neural stem and progenitor cells located in the hippocampus drive adult neurogenesis. The proliferation of these cells is essential to maintain stem cell populations and produce new neurons. A significant departure from the dogma that RA acts mainly to promote neuronal differentiation has been the finding that RA signaling increases proliferation of neural stem and progenitor cells in the adult rodent hippocampus. An increase of expression of the hypoxia-inducible factor-1α (HIF1α) and its transcriptional target vascular endothelial growth factor-A (VEGFA) mediate the proliferative effect of RA [28]. This observation broadens RA’s function beyond its well-described role in neuronal differentiation.

#### 3.2.3. Retinoic Acid Induces Stemness Rather Than Differentiation in Stem-Like Glioma Cells

In cells that display stem-like properties isolated and expanded from different gliomas, RA exerted, in general, a proproliferative and prosurvival effect mediated by RARα and RARγ [29]. Only one glioblastoma multiforme derived cell line (T1338), and a subpopulation of another (T1389), displayed neural differentiation in response to RA [29]. Since SOX2 is a master regulator of stemness in stem-like glioma cells, the effect of RA on SOX2 expression has been determined. Treatment with 1 μM RA almost abrogated SOX2 expression in T1338, where RA displayed neural differentiation, but increased SOX2 levels in T1440, T1452, and T1464, where RA exerted a proproliferative and prosurvival effect [29]. 

### 3.3. Retinoic Acid Induces Stemness or Differentiation in the Hematopoietic System

#### 3.3.1. Retinoic Acid Prevents Differentiation of Dormant Primitive Hematopoietic Stem Cells and Induces Differentiation of More Mature Blood Cells

In the hematopoietic system, RA prevents differentiation of primitive stem cells into a more mature population [84], and the opposite occurs in more differentiated blood cells and certain leukemias, in which RA displays prodifferentiation effects [85,86]. Hematopoietic stem cells are unique in their capacity to self-renew and replenish the entire blood system upon transplantation. Hematopoietic stem cells give rise to a pool of multipotent progenitors, which generate lineage-restricted progenitors, and finally, mature effector cells. A subpopulation of hematopoietic stem cells called dormant stem cells is characterized by an extremely low in vivo proliferation history with only approximately five cell divisions per lifetime in a healthy mouse [30]. These cells represent a small fraction of the hematopoietic cells in the murine bone marrow but harbor the highest long-term reconstitution potential and are reversibly activated in response to stress signals. High expression of a RA program is characteristic of dormant hematopoietic stem cells. Treatment with RA significantly attenuates the c-Myc upregulation and antagonizes stress-induced activation of these cells [30]. 

#### 3.3.2. Variable Effects of RA on Tumor Immunosuppression

Immature myeloid cells play an essential role in tumor-induced immunosuppression. These cells accumulate in large numbers in tumor-bearing hosts and directly inhibit T-cell functions. In vivo administration of RA dramatically reduced the presence of immature myeloid cells in all tested tumor models, inducing their differentiation into mature dendritic cells, macrophages, and granulocytes [87]. However, aldehyde dehydrogenase expression and the subsequent production of retinoic acid by dendritic cells, macrophages, eosinophils, and epithelial cells, seems essential in regulatory T cell induction. Regulatory T cells promote immune tolerance to tumor cells in multiple types of cancer [88].

### 3.4. RARγ Inhibits Colorectal Cancer Tumorigenesis and Metastasis, Restricting the YAP Signaling Pathway

We have observed that RA activates the YAP signaling pathway in triple-negative MDA-MB-231 breast cancer cells, but inhibits this pathway in triple-negative MDA-MB-468 breast cancer cells [25]. In vitro and in vivo studies showed that silencing RARγ expression enhanced colorectal cancer cell growth significantly, with increased migration, invasion, and metastasis, whereas ectopic expression of RARγ did the opposite, suggesting that RARγ functions as a tumor suppressor in colorectal cancer [32]. RARγ interacts with YAP in the cytoplasm of colon cancer cells and the interaction between RARγ and YAP could be significantly enhanced after RA treatment. RARγ promotes the binding of the Lats1 kinase to YAP and its phosphorylation. Phosphorylated YAP is retained in the cytoplasm, and the YAP-TEAD transcriptional activity is inhibited. YAP acts as an oncogenic regulator for cancer development. Increased expression and activity of YAP is associated with the growth, metastatic potential, and poor prognosis of several cancer types, including liver cancer and colorectal cancer [89,90,91]. 

### 3.5. Cytoplasmic Accumulation of RARγ in Hepatocellular Carcinoma Cells Plays an Oncogenic Role Via Nongenomic Activation of Akt-NFκB Signaling

Levels of RARγ were significantly elevated in tumor tissues from a majority of human hepatocellular carcinoma and in hepatocarcinoma cell lines. Overexpression of RARγ promoted colony formation by hepatocarcinoma cells in vitro and the growth of hepatocarcinoma xenografts in animals [33]. In HepG2 cells, transfection of RARγ enhanced, whereas downregulation of RARγ expression by siRNA impaired, the effect of RA on inducing hepatocarcinogenesis. RARγ interacts with the p85α regulatory subunit of phosphatidylinositol 3-kinase (PI3K). The interaction between RARγ and p85α resulted in activation of Akt and NF-κB, critical regulators of the growth and survival of cancer cells [33]. 

### 3.6. Retinoic Acid Induces Cell Differentiation and Reduces Stem Cell Markers in Pancreatic Cancer Cells

Both RA and vitamin A concentrations are reduced in pancreatic ductal adenocarcinoma tissue compared to their normal counterparts, and the expression of *RAR α* and *β*, as well as *RXR α* and *β*, are down-regulated [92]. This reduced expression of retinoid receptors correlates with a reduction of the expression of some markers of differentiation such as carbonic anhydrase II and downregulation of E-cadherin expression involved in epithelial-to-mesenchymal transition [92]. 

Herreros-Villanueva et al. [93] using several pancreatic cancer cell lines, reported that RA treatment reduced the sphere-forming capacity as well as the size of spheres formed and the expression of pancreatic stem cell markers CD24, CD44, CD133, and aldehyde dehydrogenase 1. Essential stemness genes, such as *SOX2* decreased. Surprisingly, however, the expression of the proto-oncogene c-Met was significantly increased in all the pancreatic cancer cell lines studied.

**Figure 3:**
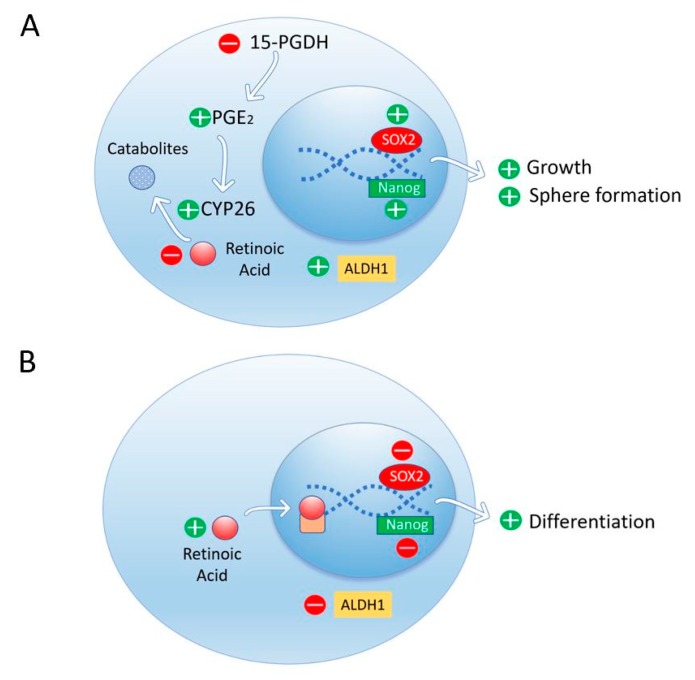
Depletion of RA by an increase of CYP26 expression due to an accumulation of prostaglandin E2 (PGE2) results in high levels of ALDH1, Sox2, and Nanog in tumor cells with growth and sphere formation (**A**). Retinoic acid replacement suppresses ALDH1 signaling in pancreatic ductal adenocarcinoma and induces cell differentiation (**B**).

The accumulation of prostaglandin E2 (PGE_2_) by inhibition of the degradation enzyme 15-hydroxyprostaglandin dehydrogenase (15-PGDH) induces progression of pancreatic ductal adenocarcinoma (PDAC) [34]. Genetic deletion of 15-PGDH showed PGE_2_ accumulation, enhanced CYP26A1 expression, and in consequence, RA depletion in the pancreas. RA depletion results in PDAC with high levels of Aldh1, Sox2, and Nanog in tumor cells, with growth and sphere formation. RA replacement suppresses Aldh1 signaling in tumor cells and tumor progression in pancreatic adenocarcinoma (Figure 3). 

### 3.7. Retinoic Acid Downregulates ALDH1-Mediated Stemness and Inhibits Tumor Formation in Ovarian Cancer Cells

ALDH1 activity is positively correlated with stemness in ovarian cancer cells according to measures such as sphere formation and stem cell marker expression, as well as tumorigenesis in a mouse xenograft model. Retinoic acid reduced ALDH1 expression, suppressed tumor formation, and inhibited sphere formation, cell migration, and invasion in ALDH1-abundant ovarian cancer cells [94].

### 3.8. Retinoic Acid Induces Cell Differentiation and Proliferation During Spermatogenesis

**Figure 4:**
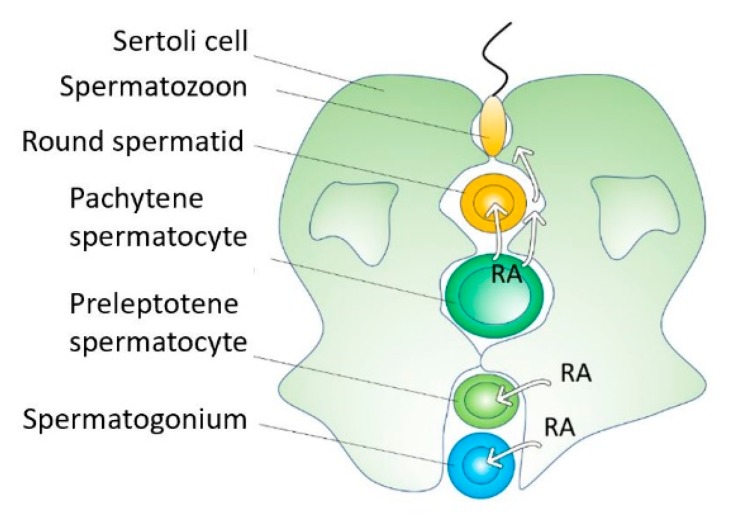
During spermatogenesis, RA controls spermatogonial proliferation and differentiation, meiotic initiation, spermatid elongation, and sperm release. Spermatogonial stem cells undergo self-renewal or proliferate and differentiate to enter meiosis in response to RA.

Cell differentiation during spermatogenesis involves four transitions: spermatogonial differentiation, meiotic initiation, spermatid elongation, and sperm release. Retinoic acid induces all four transitions [95] (Figure 4). Retinoic acid from Sertoli cells induces the premeiotic transitions. Once germ cells enter meiosis, pachytene spermatocytes produce RA to coordinate the two postmeiotic transitions [95]. Retinoic acid levels fluctuate in the testis to regulate the different transitions during spermatogenesis [95]. 

Spermatogonial stem cells remain as stem cells (self-renewal) or proliferate and differentiate to entering meiosis in response to RA. A gene induced by RA, Stra8 (stimulated by retinoic acid 8), induces spermatogonial differentiation, meiotic initiation, and proliferation of both spermatogonial stem cells and induced differentiating spermatogonia [35]. 

A fraction of spermatogonia undergoes neither spermatogonial differentiation nor meiotic initiation in response to RA, ensuring that a reservoir of undifferentiated spermatogonia is maintained throughout the animal’s reproductive lifetime [96]. Spermatogonia and their precursors prospermatogonia exhibit a different capacity to respond to RA with at least two underlying causes. First, progenitor spermatogonia are prevented from responding to RA by the catabolic activity of CYP26 enzymes. Second, a smaller subset of undifferentiated spermatogonia enriched for spermatogonial stem cells exhibits catabolism-independent RA insensitivity [97]. Retinoic acid receptor α balances proliferation and differentiation of spermatogonia, and controls genome integrity during meiosis, coordinating proper spatial and temporal development of germ cells throughout spermatogenesis [98]. 

### 3.9. Retinoic Acid Controls the Regeneration of Tissues in the Adult Organism

Retinoic acid coordinates salamander limb regrowth after amputation [99]. Recently, Kim et al. reported a mechanism of tissue regeneration in adult organisms consisting of activation of the damage sensor TLR3 receptor and the consequent induction of intrinsic synthesis of RA [36]. Using an adult model of regeneration, where stem cells regenerate de novo hair follicles after a skin injury, they propose the following hypothesis: damage of tissue might induce the release of double-stranded RNA (dsRNA) that activates the TLR3 receptor and its downstream pathways STAT3 and NF-kB. Both pathways induced the expression of aldehyde dehydrogenase1A3 (ALDH1A3), which converts retinaldehyde to RA [100]. Either RA or dsRNA, but particularly both of them together, robustly increased ALDH1A3 protein expression [36]. Under these conditions, maximal upregulation of stem cell markers and downregulation of differentiation markers occur. Retinoic acid receptors (RAR) are essential in responding to RA and enhancing regeneration. In both, humans and mice, the dsRNA-RA axis is a conserved pathway for promoting regeneration [36]. An interesting question is whether a similar mechanism can activate tumor regeneration since TLR3 stimulation facilitates stem cell-like phenotypes in breast cancer [101,102,103,104].

## 4. Conclusions 

The balance between self-renewal and differentiation of stem cells is crucial for development, regeneration in the adult organism, and cancer progression. Cancer progression involves a gradual loss of the differentiated phenotype and the acquisition of progenitor and stem cell-like features [105]. Retinoic acid is a well-known inductor of cell differentiation in many experimental models and has been effectively used in the treatment of acute promyelocytic leukemia. However, our present knowledge broadens the function of RA to include induction of cell stemness and progenitor cell proliferation. In consequence, RA can produce protumorigenic and anti-tumorigenic effects in different cancer cell types. The antagonistic effects of RA are not surprising since RA can activate more than one thousand different genes in two lines of triple-negative breast cancer cells [24]. RA also can promote extragenomic actions [23]. Moreover, the effect of RA on tumor stromal cells can shape the tumor microenvironment favoring tumor growth and invasion [74]. Finally, RA controls tumor immunosuppression in opposing ways, reducing immature myeloid cells and inducing regulatory T cells [87,88].

We need to know the critical signaling pathways controlled by RA that determine the final balance to stemness or differentiation. The main goal is to find cancer therapies able to block stemness and promote cell differentiation. Among the pathways promoting stemness controlled by RA that could be particularly significant are the VEGF signaling pathway [26,28,68,69], the Src-YAP-IL6 axis [25], and the activation of sensors of cell damage, such as TLR3 [36].

Retinoic acid controls the beginnings, transitions, and endings. With his two faces, RA looks to the past (stemness) and the future (differentiation), deciding the cell fate. 

## Figures and Tables

**Table 1:** Induction of stemness or cell differentiation by retinoic acid (RA) in a cell-type-dependent manner.

Cell Type	Action	Signaling Pathway	RA Dose-Time	References
Pluripotent stem cells	Stemness	Inhibition of Wnt. Activation of Akt-mTOR	0.5 µM (24 h)	[22]
Breast cancer cells T47D^403^	Stemness	Lack of expression of RARα tumor suppressor genes and activation of RARα-PI3K-AKT	1 µM (72 h)	[23]
Breast cancer cells MDA-MB-231	Stemness	Upregulation of 1286 genes, among them *MUC4*. Activation of the axis Src-YAP-IL6	0.1 µM (18 h) 5 µM (48 h)	[24] [25]
Breast cancer cells MDA-MB-468	Differentiation	Upregulation of 1358 genes, among them *HOXA1* Inhibition of the axis Src-YAP-IL6	0.1 µM (18 h) 5 µM (48 h)	[24] [25]
Breast cancer cells MCF-7	Stemness	Activation of ALDH1A1-HIF1α-VEGF	1 µM (48 h)	[26]
Mammary MCF12A cells and T47D breast cancer cells	Differentiation	RARβ/TET2-miR200c-Suppression of PKCζ	1 µM (24 h)	[27]
Adult hippocampus	Stemness	Activation of HIF1α-VEGF	1 µM (24 h)	[28]
Glioblastoma T1440, T1452 and T1464	Stemness	Increased SOX2 expression	1 µM (7d)	[29]
Glioblastoma T1338	Differentiation	Decreased SOX2 expression	1 µM (7d)	[29]
Dormant hematopoietic cells	Stemness	Attenuation of C-MYC expression	5 µM (24–48 h)	[30]
Hematopoietic stem cells	Differentiation or stemness	Differentiation through RARα Stemness through RARγ NOTCH1 expression	1 µM (14d)	[31]
Colorectal cancer cells	Differentiation	RARγ-inhibition of YAP-increased E-cadherin expression	1 µM (30 min)	[32]
Hepatocelular carcinoma cells	Stemness	RARγ-PI3K-AKT-NFκB	1 µM (48 h)	[33]
Pancreatic ductal adenocarcinoma	Differentiation	Decrease ALDH1, SOX2 and NANOG	10 μM (48 h)	[34]
Spermatogonial stem cells	Differentiation	Upregulation of STRA8, AGPAT3, FAM57A, WDR91	0.1 μM (24 h)	[35]
Regeneration of keratinocytes	Stemness	TLR3-STAT3 and NFkB-ALDH1-RA-RAR	0.1µM (48 h)	[36]
